# Editorial

**Published:** 1872-01-01

**Authors:** 


### MEDICAL EXAMINER.

*A Semi-Monthly Journal of Medical Sciences.*

EDITED BY

N. S. DAVIS, M. D., AND F. H. DAVIS, M. D.

Chicago, January 1st, 1872.

### EDITORIAL.

THE MEDICAL EXAMINER.— The present number of the EXAMINER is presented to its readers in a new form and new dress, and is intended to mark a new era in the progress of medical journalism in the North-west — or, rather, in the center of this great continent.

For some months before the recent great fire in this city, we had been maturing our arrangements for commencing the new volume with an enlarged sheet, a more frequent issue, and such a corps of regular correspondents as would enable us to make the EXAMINER a faithful representative of the profession throughout the whole northern part of the great interior valley of this continent. These arrangements were only temporarily interfered with by the fire, and though compelled to make our first issue a few days after the proper date, we enter upon our work confident that we shall make our journal a useful and welcome visitor to the thousands of busy practitioners throughout the country. By issuing a larger page, with double columns, on the first and fifteenth of every month, we shall give our readers a greater amount of reading matter in the year, and bring it to their attention at shorter intervals. We shall give more space than heretofore to the clinical material furnished by the several hospitals, dispensaries, etc., within our reach; to the doings of medical societies, and to the collation of items of direct practical value from the current medical literature, both domestic and foreign. In the editorial columns proper, we shall continue to notice and advocate all measures calculated to promote the social, educational and scientific interests of the profession, regardless of particular schools, booksellers, or drug-manufacturers. With ample experience in medical journalism, independent in pecuniary resources, we are under no supervision or restraints, except such as are imposed by a love of the profession and a sincere desire to advance its interests and promote its usefulness. We earnestly invite contributions from members of the profession, both in city and country, and respectfully ask a continuance of the confidence and liberal patronage which we have heretofore received. All letters and communications should be addressed to the "Publishers of the MEDICAL EXAMINER, No. 797 Wabash Av., Chicago, Ill."

SMALL-POX.—This disease appears to have prevailed so extensively in different cities and parts of the country during the last few months as to indicate a strong epidemic tendency. At the present writing there are something more than 100 cases of all grades (many light varioloid, others severe and unmodified) in this city. Reliable statements of its prevalence here are made in the weekly reports of the Board of Health; and all rumors which magnify its prevalence beyond these statements are exaggerations.

FRATERNAL.—A few days since we had the pleasure of a visit from Dr. Stone, of St. Paul, Minn., editor of the *North-western Medical Journal.* He has our best wishes for his success and prosperity.

PRIZE ESSAYS.—It is to be hoped that the Alumni of the Chicago Medical College will not forget the prize of $100 offered by the Alumni Association for the best essay on some medical subject, to be awarded by a committee at the next annual meeting. All such essays should be forwarded to the Secretary of the Association or to some member of the committee on or before the 20th day of February.

The committee to make the award consists of Drs. H. A. Johnson, E. Andrews, Walter Hay, and N. S. Davis.

No accomplishment is more valuable to the physician than facility and correctness in writing, and it can only be acquired by diligent practice.

Our City Subscribers.—We have a limited number of extra copies of the Medical Examiner for 1871, and we will cheerfully supply those of our subscribers who may have lost their numbers in the great fire *free of charge*, if they will call at our office for them.

Minnesota State Medical Society.—This active and efficient medical organization holds its next annual meeting at St. Paul, commencing February 6th, 1872. We think one or more delegates were appointed by the Illinois State Medical Society to attend the meeting of the Minnesota Society, but we have not the record before us. Dr. Franklin Staples, of Winona, is president, and informs us that any delegates from the Illinois Society will receive a most cordial welcome.

